# Influence of Temperature on Corrosion Behavior of 2A02 Al Alloy in Marine Atmospheric Environments

**DOI:** 10.3390/ma11020235

**Published:** 2018-02-03

**Authors:** Min Cao, Li Liu, Lei Fan, Zhongfen Yu, Ying Li, Emeka E. Oguzie, Fuhui Wang

**Affiliations:** 1 Corrosion and Protection Division, Shenyang National Laboratory for Material Science, Northeastern University, NO. 3-11, Wenhua Road, Heping District, Shenyang 110819, China; mcao12s@imr.ac.cn (M.C.); zfyu12s@imr.ac.cn (Z.Y.); fhwang@imr.ac.cn (F.W.); 2 Institute of Metal Research, Chinese Academy of Sciences, Wencui Road 62, Shenyang 110016, China; lfan10s@imr.ac.cn (L.F.); liying@imr.ac.cn (Y.L.); 3 Electrochemistry and Materials Science Research Laboratory, Department of Chemistry, Federal University of Technology Owerri, PMB 1526 Owerri, Nigeria; oguziemeka@yahoo.com

**Keywords:** 2A02 Al alloy, NaCl deposit, marine atmospheric corrosion, temperature

## Abstract

The corrosion behavior of 2A02 Al alloy under 4 mg/cm^2^ NaCl deposition at different temperatures (from 30 to 80 °C) has been studied. This corrosion behavior was researched using mass-gain, scanning electron microscopy-SEM, laser scanning confocal microscopy-LSCM, X-ray photoelectron spectroscopy-XPS and other techniques. The results showed and revealed that the corrosion was maximal at 60 °C after 200 h of exposure. The increase of temperature not only affected the solubility of oxygen gas in the thin film, but also promoted the transport of ions (such as Cl^−^), and the formation of protective AlO(OH), which further affects the corrosion speed.

## 1. Introduction

Atmospheric corrosion results from chemical or physical reactions between a material and the surrounding atmosphere, and is one of the most widely studied topics in the field of corrosion. The corrosion of the Al alloy has been investigated in several studies. However, most of these studies were conducted in a laboratory environment [1,2,3,4,5,6,7,8,9,10,11,12,13] due to the rapid results and economic efficiency provided by accelerated tests. It was found that serious marine atmospheric corrosion was caused by the combined action of multifactor conditions (such as sunshine, temperature, and rain) on the metal surface [14]. The marine atmospheric environment is characterized by permanently high temperatures (from sunshine) and relatively high humidity with considerable NaCl precipitation. The corrosion of materials, therefore, is accelerated by the high levels of humidity, temperature, and salinity in the marine atmospheric environment.

Al alloys are extensively employed as structural materials in the fields of transportation, electrical engineering, and aerospace applications [15,16,17]. For example, 2A02 Al alloy is commonly used as an engine fan blade material for marine serving aircraft and becomes exposed to harsh corrosive conditions in the marine environment when the aircraft is in service. Therefore, it is necessary to determine the corrosion mechanism of the alloy in such environments, and to develop effective corrosion control strategies. One of the unique features that will influence corrosion mechanisms during long-term service is the formation of a deposited salt layer on the surface of the alloy, which aggravates corrosion. This salt layer must, therefore, be taken into consideration in simulating corrosion in marine atmospheric environments.

The atmospheric corrosion behavior of Al alloys in various environments has mainly been investigated through field studies [18,19,20,21,22]. Compared with pure Al, Al alloys with second-phase particles exhibit improved mechanical properties but reduced corrosion resistance [23]. Al alloys can undergo different forms of corrosion, such as pitting corrosion [24], intergranular corrosion [25,26], and even exfoliation corrosion [27]. It has been accepted that the corrosion resistance of Al alloys is related to the formation of a passive oxide film, which naturally develops on the alloy surface under normal atmospheric conditions. However, halide ions, especially chloride ions (Cl^−^) in the marine atmospheric environmental attack and compromise this passive film, resulting in accelerated corrosion [28,29,30]. Abiola et al. [31] studied the effects of environmental factors on the atmospheric corrosion of metallic materials such as mild steel, and showed that Cl^−^ is the most significant factor to accelerate the corrosion rate. That is, chloride plays a critical role in localized corrosion such as pitting corrosion, intergranular corrosion, and stress corrosion cracking [32,33]. 

Al alloy corrosion in the marine atmospheric environment is not only affected by the high salt content in the environment, but also by a combination of meteorological and pollution factors [14]. Nishimura et al. [34] found that in such environments, the relative humidity and high salinity are complementary; the higher the humidity, the greater the availability of Cl^−^ to cause corrosion of the Al alloy. It is well recognized that atmospheric corrosion takes place only when the metal is wetted [35]. Wetting time is thus regarded as an important determinant of atmospheric corrosion. Wetting condition is usually generated by vapor condensation. The extent of condensation depends on the water content of the air and the temperature of the metal substrate surface. When the water content reaches the saturation level, water condenses in the form of dew. The critical role played by dew formation in the atmospheric corrosion of metals has been well documented [36].

Of the key determinants of marine atmospheric corrosion (high humidity, high temperature, and high salinity), the effects of different temperatures have been the least systematically investigated. This is despite the fact that the temperature influences the formation of water films on metal surfaces as well as the subsequent electrochemical reactions. High temperatures hinder the formation of water films [34,36], in which case the electrochemical reaction is unlikely to initiate and progress. At the same time, temperature also influences the rates and mechanisms of electrochemical reactions [37,38,39]. Han and Li [35] observed that the weight gain and maximum pitting depth of LY12 Al in 1 mg/100 cm^2^ Cl^−^ increased with rising temperature. Sharifi-Asla et al. [37] found that increasing the temperature from 25 to 85 °C led to a decrease in the resistance against localized corrosion of carbon steel in saturated Ca(OH)_2_ solution containing Cl^−^. Blucher et al. [40] reported a very strong positive correlation between temperature and the rate of NaCl-induced corrosion in humid air. Additionally, Esmaily et al. [41] reported that the temperature dependence of the atmospheric corrosion of AM50 alloy is attributed to the Al content in the alloy. Several crystalline magnesium hydroxy carbonates formed at 4 and 22 °C, but were absent at −4 °C. This indicates that temperature can remarkably influence the corrosion behavior of Al alloys. It is thus surprising that only a few studies have focused on the effects of different temperatures on corrosion, especially for specialized alloys such as 2A02 Al. This study is, therefore, focused on the influence of different temperatures on the corrosion behavior of 2A02 alloy in marine atmospheric environments.

Some recent studies have investigated long-term atmospheric corrosion of Al alloys. Sun et al. [42,43,44,45,46] investigated the mass loss and degradation of mechanical properties, including pit depth evolution, for AA2024-T4 after 20 years of atmospheric exposure. Such real-time, long-term exposure studies are surely immensely beneficial for our understanding of the mechanisms of corrosion in atmospheric environments. Nonetheless, systematic investigations in accelerated modified marine atmospheric environments significantly shorten the time interval for obtaining useful information to enable the design and implementation of effective corrosion control measures.

Accordingly, in the present study, laboratory-accelerated modified marine atmospheric corrosion experiments were designed. A special device was built to simulate a marine atmospheric environment identical to the actual service environment of an aircraft engine fan blade. The corrosion behavior of the 2A02 Al alloy was studied at different temperatures in this accelerated modified marine atmospheric environment using mass-gain, scanning electron microscopy (SEM), X-ray diffraction (XRD), energy-dispersive X-ray spectroscopy (EDS), laser scanning confocal microscopy (LSCM), electron probe X-ray microanalysis (EPMA), and X-ray photoelectron spectroscopy (XPS) techniques. The effect of temperature on the initial corrosion behavior of 2A02 Al alloy in this marine atmospheric environment is discussed based on these experimental results.

## 2. Experimental Procedures

### 2.1. Materials Preparation

The test alloy samples were produced from bare 2A02 Al alloy plates (without Al cladding), with the following chemical composition (in wt %): Si: 0.30, Fe: 0.30, Cu: 2.6, Mg: 2.0, Zn: 0.10, Mn: 0.45, Ti: 0.15, Al: 94.1. 

The material was cut into coupons with the dimensions 10 mm × 15 mm × 2 mm. Before the experiments, the samples were ground with 800 grit SiC paper, degreased and cleaned with acetone and ethanol in an ultrasonic bath, and then dried in flowing cool air. All the samples (original samples) were weighed and the surface area (S) was measured before the experiments.

### 2.2. Corrosion Testing

**Figure 1:**
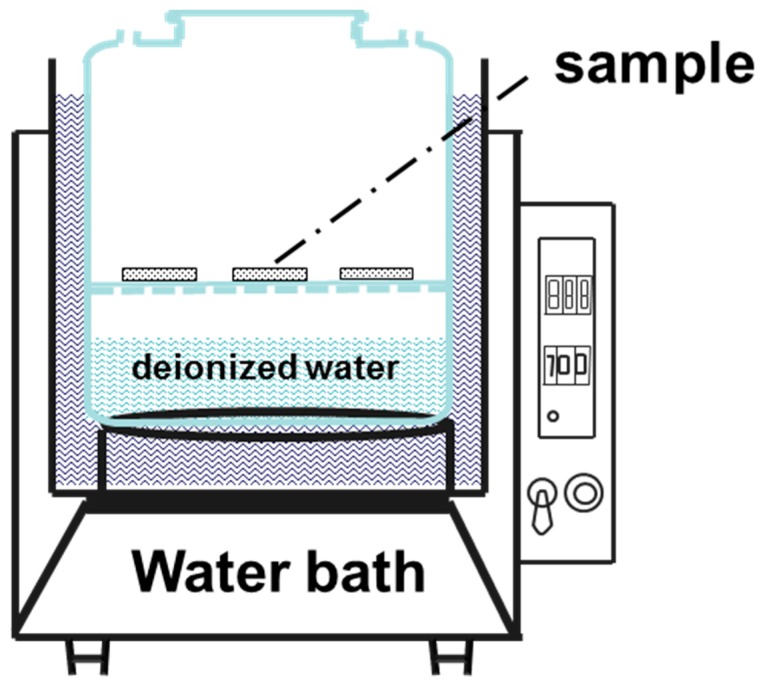
Schematic of a specially designed experimental device to simulate a modified marine atmospheric environment. It includes a water bath device to control the experimental temperature, a large glass bottle filled with deionized water, a samples holder above the deionized water in the large glass bottle. The bottleneck is small enough to prevent the evaporation of water. The most important feature is that both the sample holder and deionized water are in the temperature-controlled part of the glass bottle, which can maintain the temperature of the samples, and the deionized water is the same, and therefore, the humidity in the glass bottle can be exactly controlled.

The accelerated modified testing involved deposition of a solid NaCl layer on the surface of 2A02 Al alloy samples (not NaCl spray). The deposited NaCl layer was applied to preheat the sample surfaces by repeatedly brushing with a saturated solution of NaCl in water. The surface density of the deposited NaCl was approximately 4 ± 0.2 mg/cm^2^ [47,48]. The experimental simulation device, as illustrated in Figure 1, is divided into three parts: a water bath, a glass test container, and a perforated intermediate bulkhead. Temperature control (±0.5 °C) is achieved using the water bath, whereas the temperature in the test glass container is further calibrated by a thermometer. In order to mimic the service temperature of the aircraft engine fan blade, the selected exposure temperatures were 30, 40, 50, 60, 70, and 80 °C.

The relative humidity was maintained at 98 ± 2%, which represents an extreme humidity for atmospheric corrosion. The metal surface under this condition can form a stable NaCl solution film of thickness closely related to the environmental humidity and the surface state of the sample, and independent of temperature. The NaCl solution film thickness can be calculated using the mass of NaCl solution (*m*), surface area (*S*) and NaCl solution density (ρ):

(1) $$h=\frac{m}{S\cdot \rho }$$

According to our measurements, the thickness of the thin liquid film was 0.21 mm.

The mass gain measurements were carried out using an analytical balance with an accuracy of 0.01 mg, and the weighing at each temperature included six parallel samples. Taking the weight gain of the exposed samples and uncorroded samples into account, the actual weight gain was obtained. The corrosion products were chemically removed by pickling in the solution (50 mL H_3_PO_4_ + 20 g CrO_3_ + 1 L H_2_O) for 5–10 min at 80 °C, after which the samples were rinsed with deionized water [44]. The corrosion morphologies of the exposed samples with and without corrosion products were observed by SEM with EDS (INSPECT F50, FEI, Hillsboro, America) and LSCM (OLS4000, Olympus, Tokyo, Japan). The corrosion products were scraped off from the metallic substrate using a blade and characterized by XRD (X’Pert Pro, Pananlytital, Netherlands). A step-scanning X-ray diffractometer was used, with Cu Kα radiation, in the scanning range 10–90°. Furthermore, the elemental distribution and chemical composition of corrosion products were analyzed by EPMA (EPMA 1610, Shimadzu, Kyoto, Japan) and XPS (ESCALAB250, Thermo Fisher Scientific, Shanghai, China). EPMA equipment was a Shimadzu Model EPMA-1610 electron probe microanalyzer at an accelerated voltage of 15 kV. XPS tested powder samples that were scraped off from the metallic substrate. We chose the Al Kα radiation (1486.6 eV) as a monochromatic X-ray source. The light beam was 500 μm in diameter, with a pass energy of 50 eV. The binding energy of the carbon adsorption energy calibration was 284.6 eV.

## 3. Results

**Figure 2:**
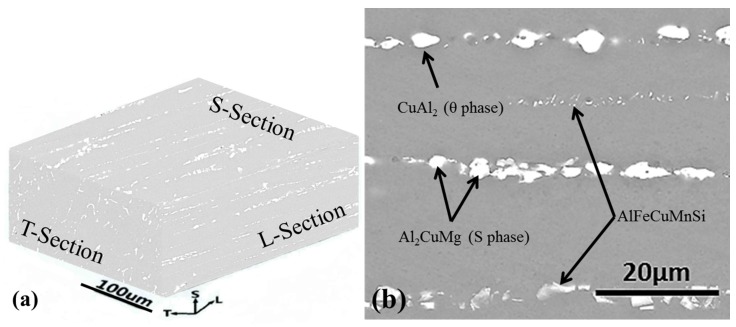
SEM morphologies of a typical microstructure of 2A02 Al alloy. (**a**) three-dimensional whole stereogram microstructure with S-section, T-section, and L-section; (**b**) detailed surface morphology of 2A02 Al alloy on S-Section microstructure.

Longitudinal (L-section), long transverse (T-section), and short transverse (S-section) are the terms conventionally used to label the three directions along the microstructure of 2A02 Al alloy. Figure 2a shows the typical microstructure of 2A02 Al alloy viewed with SEM (in backscattered electron mode), and the three-dimensional stereogram indicates the existence and distribution of constituent particles. The L-Section direction corresponds to the Al plate rolling direction, and the S-Section was the experimental side in this work. Figure 2b shows the S-Section microstructure of 2A02 Al alloy. The additional EDS results of the second phases evidenced: (i) the presence of Al, Mg and Cu elements in most of the particles; (ii) the presence of Al and Cu in the other small pellets and (iii) the presence of Al, Fe, Cu, Mn, and Si elements in some narrow strip particles. The composition of the second phases in 2XXX series Al alloys has been investigated by many researchers [49,50]. The S phase, which consists of Al, Mg, and Cu, is commonly Al_2_CuMg in 2XXX series Al alloys. The θ (CuAl_2_) phase consists of Al and Cu, and the AlFeCuMnSi phase consists of Al, Fe, Cu, Mn, and Si. In general, the second phases observed include: the Al-Cu-Mg phase identified as Al_2_CuMg (S phase), the Al-Cu phase identified as CuAl_2_ (θ phase), and the AlFeCuMnSi phase, as illustrated in Figure 1.

### 3.1. Corrosion Kinetics

**Figure 3:**
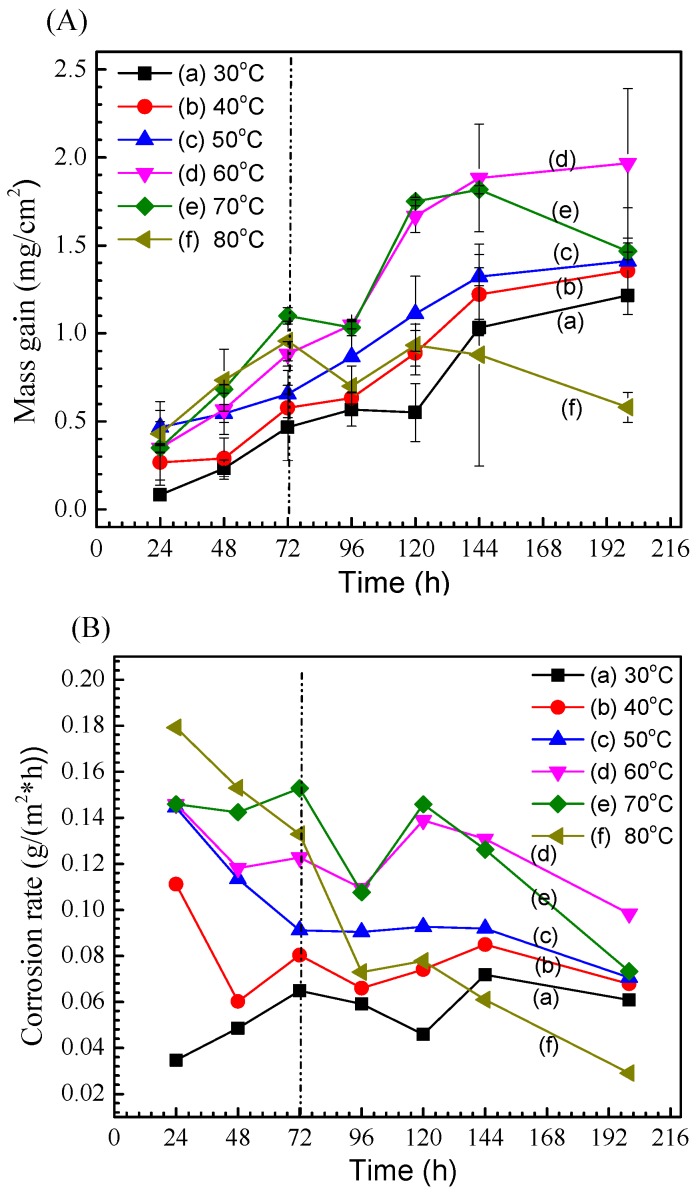
(**A**) Mass gain versus exposure time for 2A02 Al alloy exposed under 4 ± 0.2 mg/cm^2^ solid NaCl deposit in 98 ± 2% humidity at (a) 30,(b) 40, (c) 50, (d) 60, (e) 70, and (f) 80 °C; (**B**) Corrosion rate versus exposure time for 2A02 Al alloy exposed under 4 ± 0.2 mg/cm^2^ solid NaCl deposit in 98 ± 2% humidity at (a) 30,(b) 40, (c) 50, (d) 60, (e) 70, and (f) 80 °C.

Figure 3A shows the mass gain as a function of exposure time for the corrosion of 2A02 Al alloy exposed under solid NaCl deposit in 98 ± 2% relative humidity at different temperatures. It is found that mass gain continuously increased, with no pronounced restraint in the later stages at 30, 40, 50, and 60 °C. However, for 70 and 80 °C, the mass gain increases with time at the initial stage before 72 h, but exhibits a significant drop after 144 h for 70 °C and 120 h for 80 °C. Furthermore, by comparing the mass gain at different temperatures under the same exposure time, it can be seen that higher temperature can accelerate corrosion rates at the initial stages of corrosion, especially before 72 h. With prolonged exposure, this trend was only sustained at temperatures ≤60 °C, whereas the mass gain at 70 and 80 °C decreased after 72 h. Interestingly, after 200 h, the maximum mass gain was observed at 60 °C. Therefore, the corrosion behavior of this Al alloy under the test conditions manifests two distinguishing time intervals: the early stages of corrosion (1–72 h) and the later stages of corrosion (72–200 h), both of which are indicated by the dash-dot line in Figure 3A. The early stage of all the curves at different temperatures shows a trend of sustained increase in the corrosion rate; however, in the later stage, the curves present a descending trend for 70 and 80 °C. 

Figure 3B shows the oxidation weight gain per unit time, that is, the corrosion rate of 2A02 Al alloy at different temperatures and different times. It is found that the corrosion rate of 2A02 Al alloy at other temperatures decreases with time, except for 30 °C. Compared with the corrosion rate at different temperatures under the same time, it is found that the corrosion rate is similar to that in Figure 3A. Corrosion rates of 2A02 Al alloy at 30, 40, 50 and 60 °C increased with the increase of temperature at the same time. For 70 and 80 °C, the corrosion rate is also divided into two stages: in the initial stage (before 72 h), the corrosion rate increases with the increase of temperature, while in the latter stage (after 72 h), the corrosion rate at 80 °C rapidly decreased. In conjunction with the above analysis of Figure 3A,B, we chose the temperatures of 30, 60, and 80 °C to perform further detailed measurements according to the characteristics of the mass gain curves.

### 3.2. Morphology and Composition

**Figure 4:**
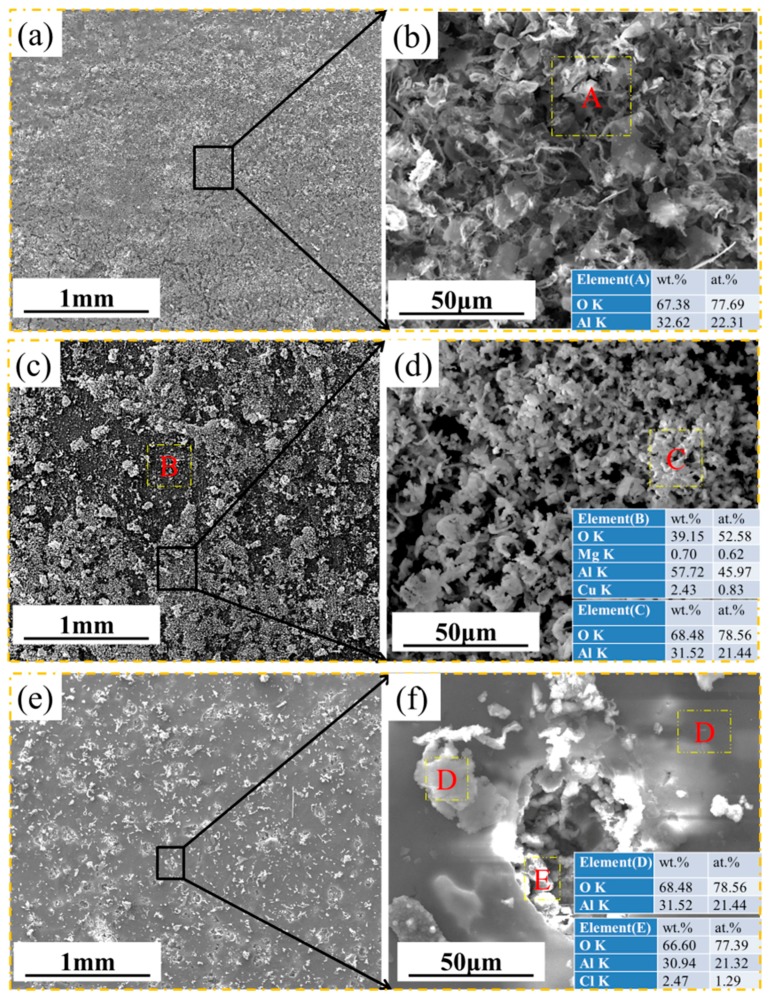
SEM images of microscopic surface morphology of the samples after corrosion 72 h under 4 ± 0.2 mg/cm^2^ solid NaCl deposit in 98 ± 2% humidity: (**a**,**b**) at 30 °C; (**c**,**d**) at 60 °C; and (**e**,**f**) at 80 °C. EDS results for A-E locations were also presented.

Figure 4 shows SEM images of the surface morphology of the samples at 30, 60, and 80 °C after corrosion for 72 h. Figure 4a shows that the corrosion products formed at 30 °C covered the entire surface of the sample, which have a clear and wood-shaving shape, as shown in Figure 4b. The EDS results show the corrosion product in region A to be rich in the elements O and Al. Figure 4c shows that the corrosion products formed at 60 °C contain a dark compact inner layer and a loose white outer layer. This white loose outer layer also has shaving-shaped particles, though significantly smaller and more fine-grained than those formed at 30 °C. The EDS results show that there are Al oxides in the outer layer, whereas the inner layer comprised a mixture of Al, Cu, Mg, and O. The morphology of the corrosion products at 80 °C, as shown in Figure 4e, is characterized by a compact layer with tiny particles scattered on its surface. However, the larger magnification image in Figure 4f gives information that the dense layer contains a lot of corrosion defects with high Cl content.

**Figure 5:**
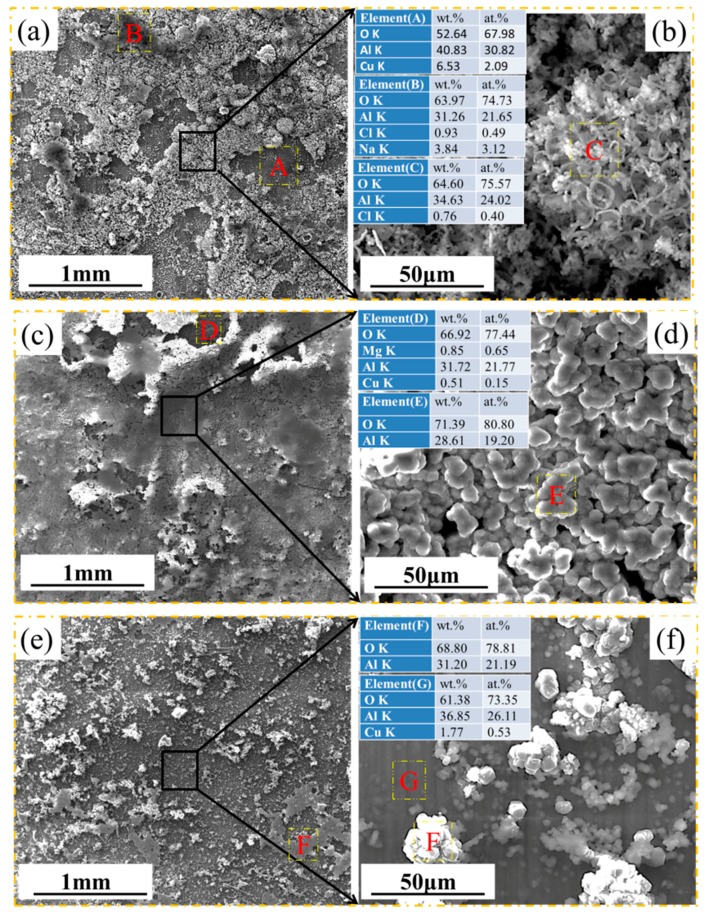
SEM images of microscopic surface morphology of the samples after corrosion 200 h under 4 ± 0.2 mg/cm^2^ solid NaCl deposit in 98 ± 2% humidity: (**a**,**b**) at 30 °C; (**c**,**d**) at 60 °C; and (**e**,**f**) at 80 °C. EDS results for A-G locations were also presented.

**Figure 6:**
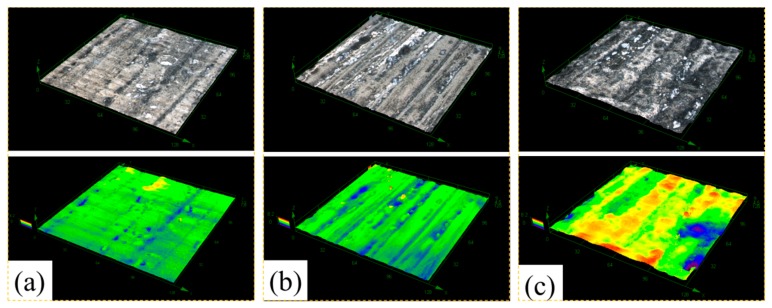
LSCM morphologies of 2A02 Al alloy substrate on removal of corrosion products after corrosion 72 h (**a**) at 30 °C; (**b**) at 60 °C; and (**c**) at 80 °C.

The surface morphologies of the corrosion scales formed on samples after corrosion at several temperatures for 200 h are shown in Figure 5. Figure 5a shows that the corrosion product formed at 30 °C comprised a dark compact inner corrosion layer and a loose white outer layer. From the enlarged photograph of the loose white outer layer in Figure 5b, wood-shaving shaped Al oxides (region C) with similar morphology to those in Figure 4b can also be found. The EDS result of region A in Figure 5a indicates that the inner layer is rich in the elements O, Al and a little Cu, whereas region B appears as flakey Al oxide containing the residual original salt. For the tests conducted at 60 °C, it can be seen from Figure 6c that the Al oxide film was more complete than that formed at 30 °C, but exhibited obvious crack detachment. The inner products contain O, Al, Mg, and Cu. The areas free of cracks are packed by lump-like-shaped particles, mainly comprising Al and O. For the corrosion scale formed at 80 °C, as shown in Figure 5e, the tiny white particles in the outer layer of Al oxide are evenly dispersed on the surface, whereas the inner compact layer contains Al, O, and Cu.

**Figure 7:**
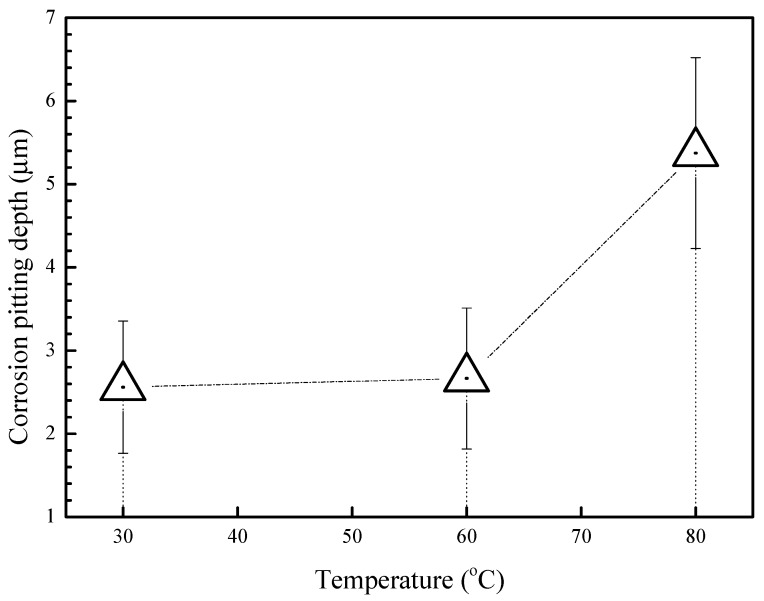
Measured results of corrosion pitting depths of 2A02 Al alloy substrate on removal of corrosion products after corrosion 72 h at 30 °C, 60 °C and 80 °C. This result corresponds to Figure 6 and was obtained from measured results by LSCM using our own software OLS4000.

In order to get a better understanding of the corrosion behavior of the 2A02 Al alloy, LSCM was used to characterize the substrate surface after removal of the corrosion products. Figure 6 shows the morphologies of 2A02 Al alloy substrates devoid of corrosion products after 72 h of corrosion at 30, 60, and 80 °C. LSCM can provide insights regarding the inner structure and the stereoscopic structure of the 2A02 Al alloy substrate. All the results are obtained under the same test conditions and displayed at the same magnification (the size is 128 µm × 128 µm) for a clear comparison. From the results, it is clear that the 2A02 Al alloy underwent localized corrosion, especially pitting corrosion. The change of color in the LSCM images indicates the pit depths. With the increase in temperature from 30, through 60, to 80 °C, it can be seen that the number of pits increased correspondingly. The pit depth was measured using the software OLS4000, all of which are shown in Figure 7. The corrosion pit depths at 30 and 60 °C were rather similar, but became more pronounced at elevated temperatures. Apparently, corrosion pits would develop at all temperatures in the range of 3–80 °C, but pit sizes and depths varied with increasing temperature.

**Figure 8:**
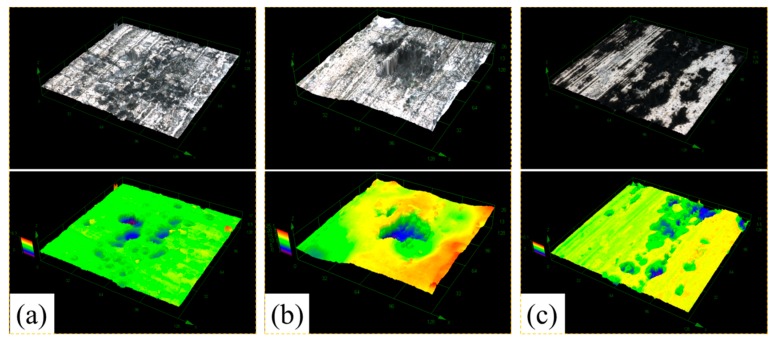
LSCM morphologies of 2A02 Al alloy substrate on removal of corrosion products after corrosion for 200 h (**a**) at 30 °C; (**b**) at 60 °C; and (**c**) at 80 °C.

**Figure 9:**
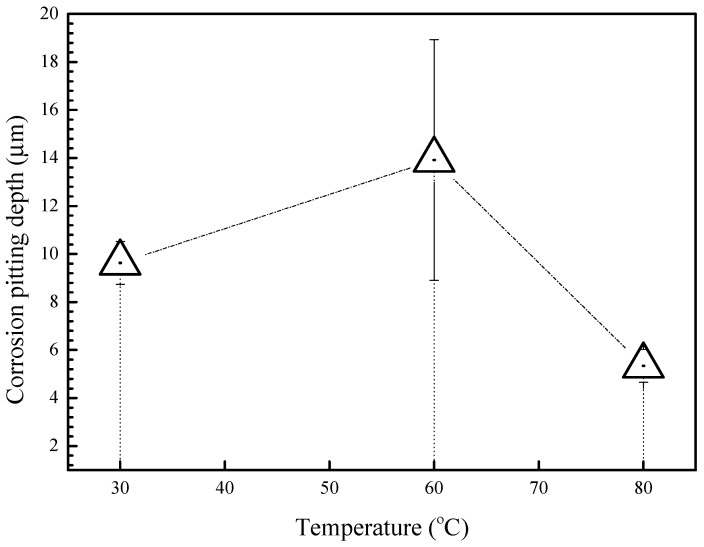
Measured results of corrosion pitting depths of 2A02 Al alloy substrate on removal of corrosion products after corrosion for 200 h at 30 °C, 60 °C and 80 °C. This result corresponds to Figure 8 and was obtained from measured results by LSCM using our own software OLS4000.

Figure 8 shows the LSCM morphologies of 2A02 Al alloy substrates without the corrosion product after 200 h at different temperatures. The results show striking deviations from those obtained after 72 h of corrosion. As shown in the results, the number of pits changed in the order: 60 °C < 30 °C < 80 °C. However, the corrosion pit depths in Figure 8 as well as the measured depth data in Figure 9 show that the deepest pits appeared at 60 °C, whereas the average value of pit depth at 30 °C is greater than that at 80 °C. In other words, the value of pit depth changed in the order: 60 °C > 30 °C > 80 °C. Indeed, the change trend of results in Figure 7 and Figure 9 are consistent with those in the mass gain curves shown in Figure 3A.

**Figure 10:**
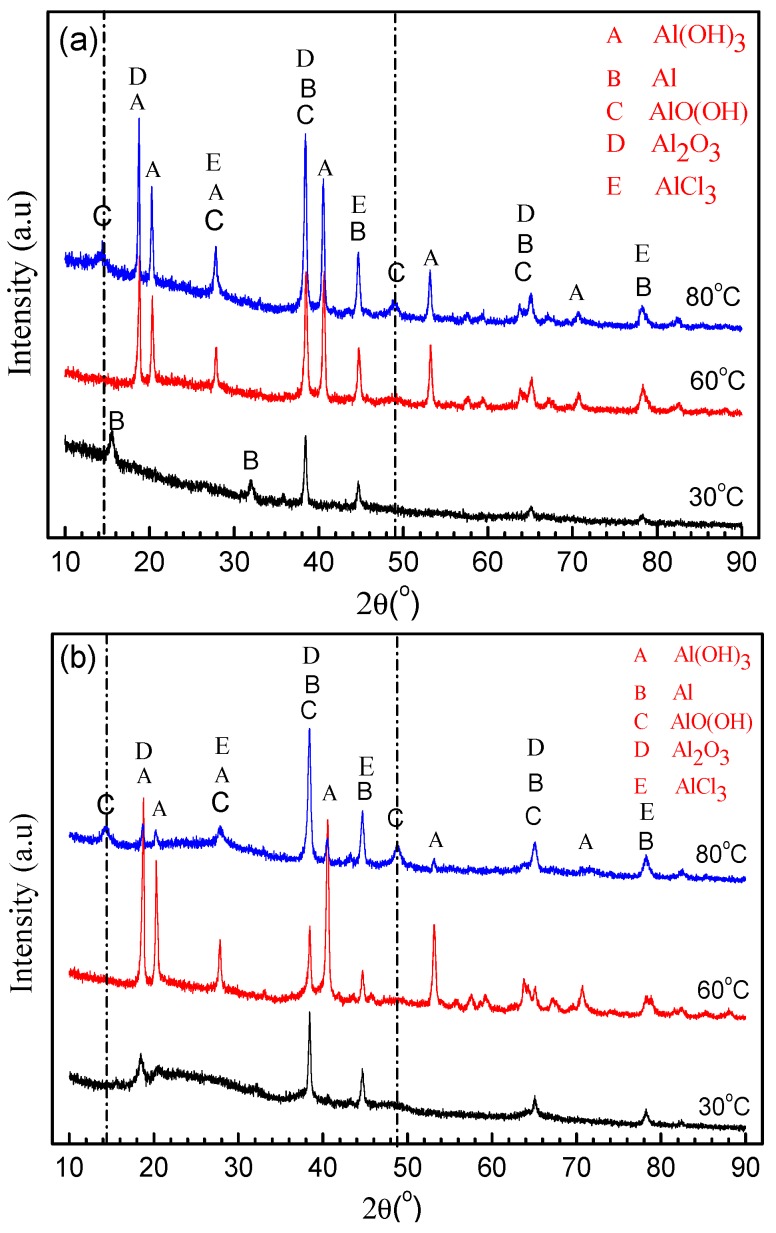
XRD patterns of the corrosion products formed on the surface of 2A02 Al alloy under 4 ± 0.2 mg/cm^2^ solid NaCl deposit in 98 ± 2% humidity, at 30 °C, 60 °C, and 80 °C: (**a**) corrosion for 72 h and (**b**) corrosion for 200 h.

Figure 10a shows the XRD patterns of the corrosion products formed on the surface of 2A02 Al alloy after 72 h corrosion at 30, 60, and 80 °C. The thickness of the corrosion scales after exposure at different temperatures, deduced from the mass gain obtained by the analysis in Figure 3, is much larger than the XRD detection depth. Therefore, corrosion products were scratched from the samples and the resulting powders were tested by XRD. According to the ICDD cards, Al(OH)_3_, AlCl_3_, and Al_2_O_3_ were present on all samples. For the results at 80 °C, two diffraction peaks around 2θ = 14° and 2θ = 49° were detected, which are ascribed to AlO(OH). It is noteworthy that AlO(OH) is only detected on the sample at 80 °C.

Figure 10b shows the XRD patterns of the corrosion products formed on the surface of 2A02 Al alloy after 200 h of corrosion. The corrosion products Al(OH)_3_, AlCl_3_, and Al_2_O_3_ can be detected on all samples corroded at different temperatures, whereas AlO(OH) was again only detected on samples corroded at 80 °C.

By comparing the XRD results for samples obtained after 72 and 200 h at 30, 60, and 80 °C, it is clear that Al_2_O_3_, Al(OH)_3_, and AlCl_3_ formed during both time intervals and at all the temperatures, but AlO(OH) was only detected at 80 °C. 

## 4. Discussion

### 4.1. Pitting Corrosion of 2A02 Al Alloy in the Accelerated Modified Marine Atmospheric Tests

The corrosion process of 2A02 Al alloy under the thin electrolyte film is subject to the general rule of electrochemical corrosion and also has the characteristics of atmospheric corrosion. The relative humidity was 98 ± 2% in this experiment, and thus a thin water film visible to the naked eye formed on the surface of all samples. The thickness of the thin electrolyte film was very thin, approximately 0.21 mm, which was calculated using Equation (1). In this case, oxygen in the air can diffuse at a high speed through the liquid film to the surface of the metal. Therefore, the overall corrosion process is a couple of reactions: the electrochemical dissolution of the Al alloy and the cathodic reduction of molecular oxygen.

**Figure 11:**
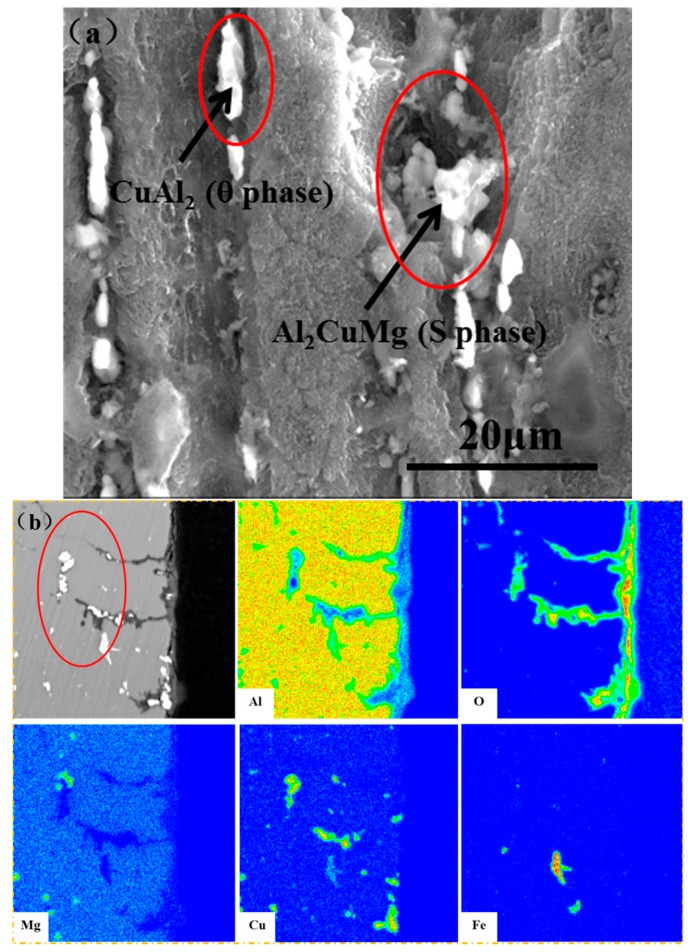
(**a**) Detailed information of pits on the 2A02 Al alloy substrate by removing of corrosion products after corrosion for 200 h under 4 ± 0.2 mg/cm^2^ solid NaCl deposit in 98 ± 2% humidity at 60 °C. This picture shows the surface morphology obtained by SEM. It shows that pits formed around the second phases on 2A02 Al alloy include Al-Cu-Mg/Al-Cu phases; (**b**) EPMA maps of the elemental distribution of the cross-section of 2A02 Al alloy under 4 ± 0.2 mg/cm^2^ solid NaCl deposit in 70% humidity at 60 °C within 200 h, the detailed information of the area of corrosion around Al-Cu-Mg/Al-Cu phases.

Figure 11a shows the morphology of the 2A02 Al alloy substrate without the corrosion product layer after 200 h of corrosion at 60 °C. From Figure 11, it is clear that the pits formed around the second-phase particles included the Al-Cu-Mg and Al-Cu phases in 2A02 Al alloy, as indicated by the red line. Corrosion mainly takes place on Al substrates around these second phase particles. The differences in chemical potentials between the Al-Cu-Mg and Al-Cu phases and the Al substrate lead to electrochemical reactions on the thin electrolyte film: these second phases function as cathodes and the Al substrate metal as the anode [46]. The electrochemical reaction accelerates the dissolution of the Al substrate around the Al-Cu-Mg and Al-Cu phases, and pitting corrosion occurs. The areas around the second phases have been found to be common sites for pit nucleation [51]. Figure 11b shows the EPMA maps of the elemental distribution of the cross-section of 2A02 Al alloy under 4 ± 0.2 mg/cm^2^ solid NaCl deposit in 70% humidity at 60 °C within 200 h. The corrosion occurs along the interface of the second-phase particle, which could be the intergranular corrosion. In this experiment, Mg is exhausted after 200 h of corrosion, as shown by the red circle in Figure 11b, where Mg in the second phase was consumed, leaving only Cu. This implies that the initial corrosion on 2A02 Al alloy should occur at the Al-Cu-Mg phase. Mg initially dissolves in the second phase, and after it is exhausted, the remaining second phase functions as a cathode phase around the dissolving Al. Some studies report that Mg is first corroded in the second phase and in some areas, local dissolution is on the threshold of pit initiation [51]. This is evidence that localized dissolution of the Al substrate initiates around the second-phase particles.

Therefore, Al-Cu-Mg and Al-Cu act as the cathode in the electrochemical reaction and the following reaction takes place:

O_2_ + 2H_2_O + 4e^−^ = 4OH^−^ (2)

The partial anodic reaction occurring at anodic sites (the place around the second phase) during the localized corrosion of Al alloys under the thin electrolyte film is:

Al = Al^3+^ + 3e^−^ (3)

Thus, as a result of the electrochemical reaction, the concentration of hydroxide ions increases in the localized corrosion sites; therefore, the local pH becomes more alkaline, and the following chemical reaction occurs [50]:

Al^3+^ + 3OH^−^ = Al(OH)_3_ (4)

Meanwhile, it has been generally acknowledged that the presence of chloride ions in an environment leads to pitting corrosion of Al alloys. Several mechanisms have been proposed to illustrate the role of chloride ions in the pitting corrosion of passivating metallic materials such as Al alloys [52,53,54].

The AlCl_3_ identified in this work provides proof that chloride ions react with cations, such as Al^3+^, to form chloride-containing compounds. Some mechanisms have been proposed to describe the formation of AlCl_3_ [55]:

Al(OH)_3_ + 3Cl^−^ = AlCl_3_ + 3OH^−^ (5)

Furthermore, according to Equations (1) and (5), the pH at the corrosion sites will become more alkaline. Therefore, we washed the sample and measured the pH of the cleaning solution by an acidometer. The measured pH was approximately 9.21, which is consistent with the expected alkaline value. NaCl is very corrosive in this environment, because Na^+^ supports the development of high pH in the cathodic areas, resulting in alkaline dissolution of the alumina passive film and rapid general corrosion.

Under some conditions, Al(OH)_3_ would further transform into Al_2_O_3_ and AlO(OH) [56]:

2Al(OH)_3_ = Al_2_O_3_ + 3H_2_O or Al(OH)_3_ = AlO(OH) + H_2_O (6)

AlO(OH) was only found at 80 °C, not 30 °C or 60 °C.

### 4.2. Effect of Temperature on the Corrosion Behavior of 2A02 Al Alloy in Accelerated Modified Marine Atmospheric Tests

It is well known that high humidity can cause liquid films to form on the surface of samples, and that the higher the humidity, the thicker the thin electrolyte film. The thickness of the thin electrolyte film, in turn, affects the diffusion of oxygen and the concentration of salt in the thin electrolyte film. However, in this 98 ± 2% relative humidity environment, salt can be fully dissolved in the liquid film; this is to ensure the samples have the same film thickness and same NaCl salinity under different temperatures. Then, the temperature is the only variable environmental factor. To get a better understanding of the influence of temperature, we discuss the effects of temperature on 2A02 Al alloy corrosion from the following perspectives.

First, the temperature can influence the diffusion of ions in the thin electrolyte film, and especially the oxygen molecules, which directly react via the cathode reaction in local corrosion. Because of the temperature changes, the oxygen solubility and oxygen diffusivity in the liquid film will change, which directly affect the electrochemical reaction of the cathode, and then affect the corrosion. The solubility coefficient *β* is affected not only by temperature but also the composition of the solution. The experimental changeable factor is temperature in the test, and others are stable. Therefore, other factors have not been discussed. According to Henry’s Law, the solubility of oxygen in the thin electrolyte film is related to oxygen partial pressure and the thin electrolyte film temperature at the gas-water interface. The higher the temperature, the higher the partial pressure of water vapor, so that the lower the partial pressure of oxygen results in a lower the content of dissolved oxygen in water. The oxygen diffusivity *C* in the thin electrolyte film can be estimated by the Arrhenius law. According to the Arrhenius law, the relationship between the oxygen diffusion coefficient and temperature *T* is *C* = *K Po*_2_ *exp*(*−β*/*T*). When the oxygen diffusion coefficient is calculated, the oxygen diffusivity *C* can be obtained. It can be seen that the diffusion coefficient increases with increasing temperature.

The cathodic reaction in Equation (2) is the rate-limiting step of this electrochemical experiment. Therefore, the dissolved oxygen solubility and oxygen diffusivity in the thin electrolyte film can significantly affect the rate of 2A02 Al corrosion. The increase in temperature causes the oxygen diffusion coefficient to increase, and the dissolved oxygen solubility decreases in the thin electrolyte film. Therefore, there is an optimal temperature (in this test it is 60 °C), at which the dissolved oxygen solubility and oxygen diffusivity are moderate, and in turn maximize the corrosion speed of 2A02 Al alloy. The mass-gain changes with temperature are well associated with the trade-off between decreasing dissolved oxygen solubility and the increasing oxygen diffusivity in the thin electrolyte film.

In addition to affecting the diffusion of oxygen in the thin film, the temperature can also affect the diffusion behavior of the metal ions, Cl^−^, or other electric-charged species in the oxide film, which further affects the corrosion speed. Therefore, this is discussed below. 

**Figure 12:**
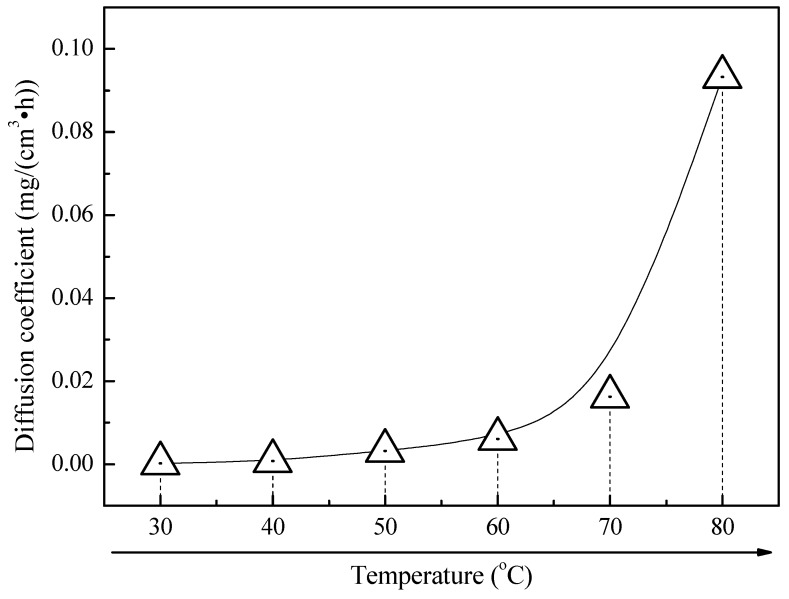
Trend chart of the relationship between the diffusion coefficient and temperature.

**Figure 13:**
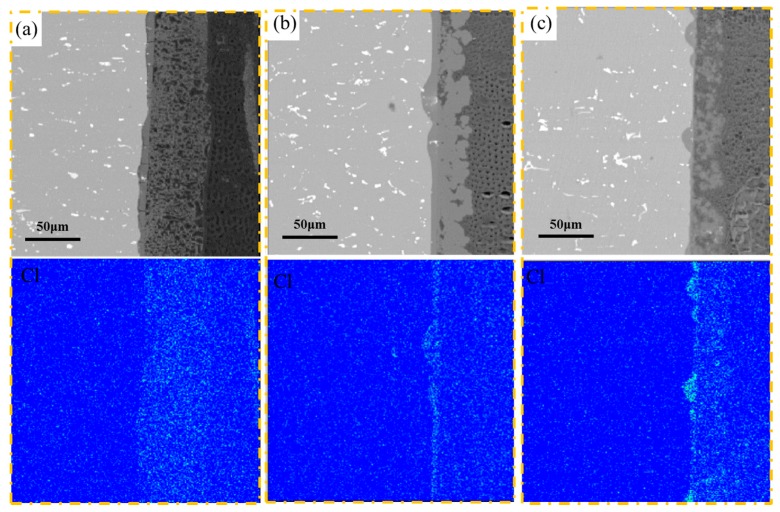
EPMA maps of the elemental distribution of the cross-section of 2A02 Al alloy after corrosion for 200 h at (**a**) 30; (**b**) 60; (**c**) 80 °C.

According to the corrosion kinetics curve of 2A02 Al alloy at different temperatures in Figure 3A, the parabolic law was used to fit the corrosion kinetics curve and then obtain the rate coefficient. The rate coefficient is equal to the diffusion coefficient under this test. Figure 12 shows the trend chart of the relationship between the diffusion coefficient and temperature. The diffusion coefficient increases with increasing temperature. Therefore, a high temperature can promote the diffusion of the metal ions, Cl^−^, and other electric-charged species. High temperatures can also promote the diffusion of the corrosive Cl^−^ in the thin liquid film to the substrate through the corrosion products, which accelerates the rate of pitting corrosion of Al alloy during the whole corrosion process. Figure 13 shows the EPMA maps of the elemental distribution in the cross-section of 2A02 Al alloy corrosion products after 200 h corrosion at 30, 60, and 80 °C. At 30 °C, there is no obvious aggregation of Cl^−^. Rather, Cl^−^ dispersed throughout the entire corrosion layer. However, an aggregation of Cl^−^ is then obvious in the inner corrosion layer near the interface between the substrate and corrosion product at 60 and 80 °C. This aggregation is more significant at 80 °C than that at 60 °C. This, once again, proves that Cl^−^ diffuses faster to the substrate with increasing temperature. Another feature of temperature in the electrochemical corrosion is that the number of the surface reactivity points increases with the increase in temperature for the electrochemical reaction on the surface of 2A02 Al alloy. More active spots thus speed up the reactions (2)–(3) and accelerate the corrosion process. Therefore, the number of pits at 80 °C is highest in Figure 8. However, the corrosion rate exhibits a decreasing trend when the temperature is higher than 60 °C (see Figure 4). This phenomenon is unexpected and inconsistent with the above temperature acceleration theory. Possible reasons to explain this slowdown are discussed below.

**Figure 14:**
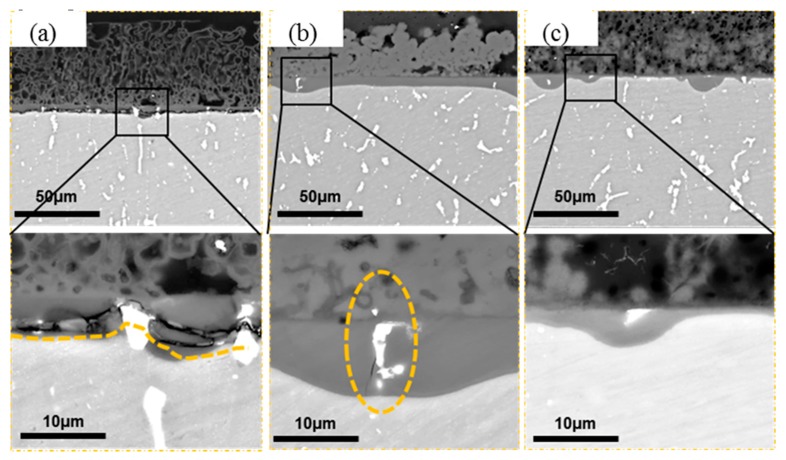
Cross-sectional morphology of the corrosion product layers formed on 2A02 Al alloy after corrosion for 200 h at (**a**) 30; (**b**) 60; (**c**) 80 °C.

Figure 14 shows the cross-sectional morphology of the corrosion product layers formed on 2A02 Al alloy after 200 h of corrosion at several temperatures. It shows that corrosion products have two layers: a loose outer layer and a compact inner layer at all three temperatures. It is interesting that the interface between both layers looks like the original base metal surface. The second phase remained in the compact inner layer of corrosion products, which seemed no longer involved in the corrosion process. The enlarged morphology images of the inner oxide layers formed at the three temperatures showed obvious cracks appearing in the inner layer at 30 °C, and a small number of cracks were found in the inner layer of the corrosion products formed at 60 °C. However, the cracks are almost unseen in the inner layer of the corrosion products formed at 80 °C. This possibly suggests that the inner layer of the corrosion products became more and more compact with increasing temperature.

**Figure 15:**
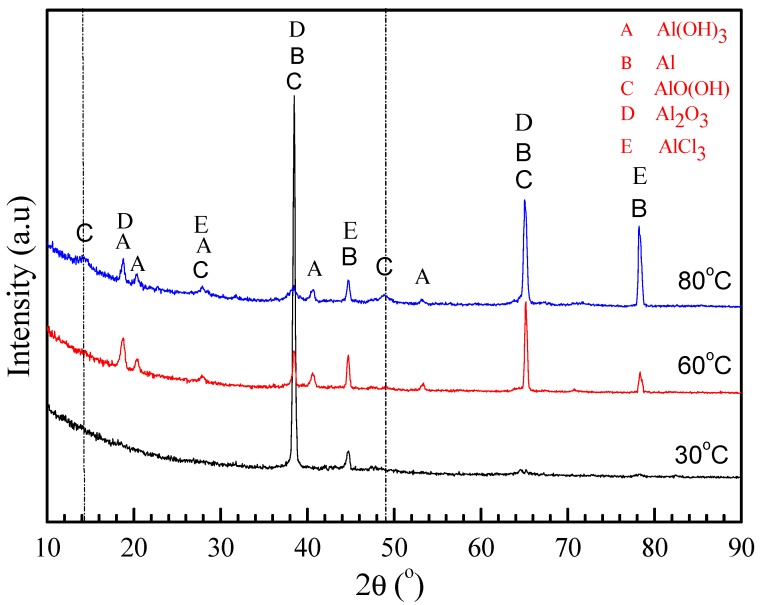
XRD patterns of the inner corrosion products formed on the surface of 2A02 Al alloy after exposure at different temperatures for 200 h.

**Figure 16:**
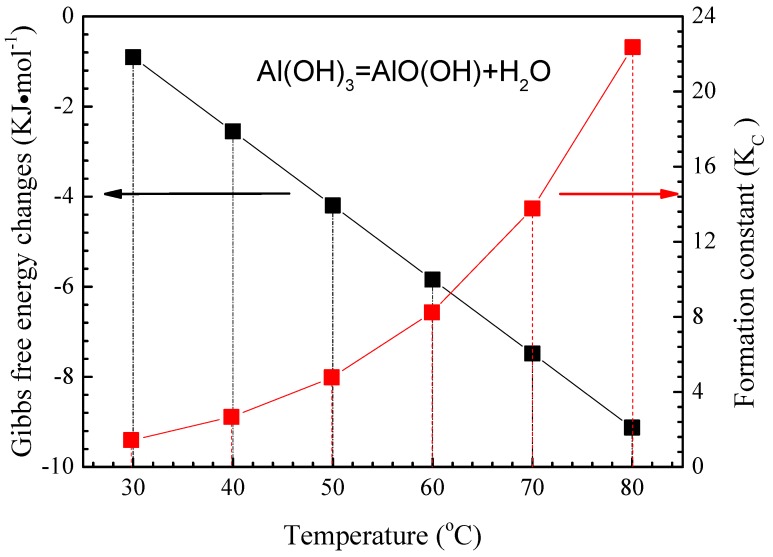
The values of reaction Gibbs free energy and formation constants for reaction (6) at different temperatures.

Figure 15 shows XRD patterns of the inner oxide layer of the corrosion products formed after 200 h corrosion at the three temperatures (polished samples with SiC paper to remove the outer corrosion layer, and verify it by SEM to observe the sample polished cross-section). It shows that AlO(OH) was only detected at 80 °C. AlO(OH) is thought to be produced by reaction (6), and the Gibbs free energy (ΔG°) vs. temperature of reaction (6) can be calculated from the relative thermodynamic data [57]. Figure 16 shows the calculated ΔG° values for reaction (6) of 2A02 Al alloy at different temperatures. This Gibbs free energy is calculated using the software HSC Chemistry 6.0, which is designed for various kinds of chemical reactions and equilibria calculations. The temperature, molar mass, and reaction (6) are written into the software and the software automatically gives the value of the Gibbs free energy and reaction constant for the reaction (6) at different temperatures. It can be seen from Figure 16 that ∆G° in reaction (6) is negative, and the higher the temperature; the more negative its value. Moreover, according to K_c_ values at each temperature, the higher the reaction temperature, the larger K_c_, and the easier the reaction to form AlO (OH). Clearly, the more negative values of ΔG° and more positive values of K_c_ at 80 °C can make the reaction (6) more easily achievable. From the calculated ΔG° values of reaction (6), it is clear that the reaction will occur when the temperature is more than 30 °C. The higher ΔG° at 30 and 60 °C means there will be lower amounts of AlO(OH) at these temperatures, thus accounting for the inability to detect AlO(OH) by XRD at 30 and 60 °C. At 80 °C, AlO(OH) is more easily formed, with higher amounts in the inner layer. We have determined that AlO(OH) can penetrate into the pores and cracks in the oxide film on the surface of 2A02 Al alloy through the thin liquid film, and then the colloidal particles close up the pores and cracks, thus slowing down the corrosion rate [58,59,60]. It is, therefore, clear that the formation of AlO(OH) can close up the inner cracks in the oxide film, thereby preventing further corrosion as observed at 80 °C.

Reactions (1)–(5) describe in detail the corrosion mechanism of 2A02 Al alloy in the simulated marine atmospheric environment. The relative compositions of the corrosion products directly reflect the reaction conditions during the test. Through the analysis of the corrosion products composition and the electrochemical reactions, we know that a protective product AlO(OH) was produced as well as the destructive product AlCl_3_. It is well known that Cl^−^ plays a key role in the chloride-induced pitting model, and that AlCl_3_ may be formed according to reaction (5). Therefore, the content of AlCl_3_ contained in corrosion products in this test reflects the degree of the pitting corrosion. Hence, by comparing the relative content of AlCl_3_ and AlO(OH) in the corrosion products of 2A02 Al alloy formed at different temperatures, we can evaluate the relative rates of the corrosion processes. 

**Figure 17:**
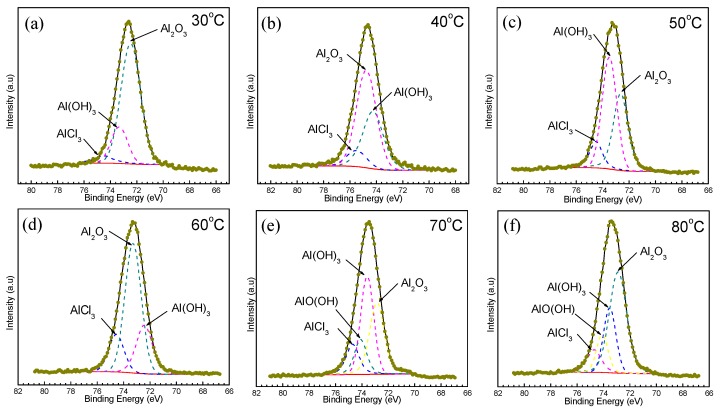
XPS result of 2A02 Al alloy prepared in the simulated marine atmospheric corrosion environment within 200 h at (**a**) 30; (**b**) 40; (**c**) 50; (**d**) 60; (**e**) 70; and (**f**) 80 °C.

Figure 17 shows the XPS results of the powder corrosion product of 2A02 Al alloy formed after 200 h of corrosion in the simulated marine atmospheric environment at different temperatures. The corrosion scales were scraped off from the corroded samples of 2A02 Al alloy. The powder samples could subsequently be comprehensively characterized to provide information about the composition of the corrosion layer. Through the XPS-peak-differentiation of Al at different temperatures, we detected the presence of Al_2_O_3_, Al(OH)_3_, and AlCl_3_ formed at all the temperatures, but AlO(OH) only appeared at 70 and 80 °C. This result is consistent with the XRD analysis. In XPS analysis, all the possible compounds containing Al were deduced from the XPS-peak-differentiation, and the peak area of the possible compound represents its relative content in the corrosion products. However, it should be noted that the relative content of different compounds containing the same element can only be compared in the same sample. In order to give a clearer description about the content of the corrosion products, the intensity curves in the XPS results were processed to be displayed numerically. The compound atomic percentage, which is represented by the XPS-peak-differentiation area percentage, was used to express the content of the corrosion products. The ratio of the AlCl_3_ peak area to that of the total Al is regarded as the content percentage of AlCl_3_ in the sample, as shown in Figure 17. The content percentages for other compounds containing Al were similarly obtained. The calculated contents of each compound produced at different temperatures are shown in Table 1.

The relative content of AlCl_3_ in the corrosion products increased with temperature up to 60 °C. As we know that AlCl_3_ was produced in the process of electrochemical corrosion (Equation (5)), the involvement of Cl^−^ as a reactant in Equation (5) is the precondition for pitting corrosion, so the amount of AlCl_3_ can be used to evaluate the degree of corrosion. The higher the amount of AlCl_3_, the more reaction (5) may occur, and the more serious the pitting corrosion. Meanwhile, we know the relatively high corrosivity of AlCl_3_ is explained by the formation of an acidic surface electrolyte and by the high solubility of aluminum hydroxy chloride [61,9]. Therefore, the presence of AlCl_3_ can accelerate the corrosion of 2A02 Al alloy. The relative content of Al_2_O_3_ in corrosion products decreased at first and then increased with increasing temperature, reaching a minimum at 60 °C. Al_2_O_3_ has stable chemical performance, excellent dielectric properties as well as resistance to chemical corrosion. The dense Al_2_O_3_ layer thus possesses good corrosion protection properties. The relative content of Al(OH)_3_ in corrosion products increased at first and then decreased with increasing temperature. The combined XPS results of AlCl_3_ and Al_2_O_3_ clearly show that the corrosion susceptibility of 2A02 Al was highest at 60 °C. AlO(OH) was mainly detected at 70 and 80 °C, and has been shown to penetrate and seal up the pit pores, to form a corrosion-resistant layer [58]. According to cross-sectional morphologies of the corrosion products in Figure 14, we can also see that the corrosion products have a more compact structure at the interface between the corrosion products and base metal. 

## 5. Conclusions

In this study, effect of temperature on corrosion behavior of 2A02 Al alloy in a simulated marine atmospheric environment has been systematically investigated. It is found that high temperatures not only decrease the dissolved oxygen solubility and increase the oxygen diffusivity in the liquid film, but also promote the transport of ions (such as Cl^−^) and accelerate the formation of Al corrosion products. The formation of AlO(OH) can make the inner layer of the passive film more compact and ultimately hinders further corrosion.

Effect of temperature on the corrosion mechanism is elucidated as follows. At low temperature (<60 °C), the slower Cl^−^ diffusion, low content of AlCl_3_ and AlO(OH) and high content of Al_2_O_3_, make the corrosion rate very low. At high temperature (>60 °C), while the diffusion of Cl is fast, the higher AlO(OH) content causes the inner corrosion layer to be significantly protected, and thus reduces corrosion rate. Therefore, there is a middle temperature, 60 °C, which has quick Cl diffusion and less AlO(OH) content; hence, the corrosion rate of 2A02 Al alloy at this temperature is maximal.

## Figures and Tables

**Table 1:** The contents of each compound produced at different temperatures (at %).

Contents	30 °C	40 °C	50 °C	60 °C	70 °C	80 °C
$\frac{AlCl_{3}}{Al_{all}}$	0.06	0.09	0.12	0.16	0.14	0.11
$\frac{Al_{2}O_{3}}{Al_{all}}$	0.75	0.56	0.39	0.22	0.35	0.53
$\frac{AlOOH}{Al_{all}}$	0	0	0	0	0.15	0.13
$\frac{Al{\left(OH\right)}_{3}}{Al_{all}}$	0.19	0.35	0.49	0.62	0.36	0.23
